# Supplementary material for: Molecular Markers for Detecting Inflorescence Size of Brassica oleracea L. Crops and B. oleracea Complex Species (n = 9) Useful for Breeding of Broccoli (B. oleracea var. italica) and Cauliflower (B. oleracea var. botrytis)
Source: Plants (Basel). 2023 Jan 15;12(2):407. doi: 10.3390/plants12020407 (PMC9862391; doi:10.3390/plants12020407)
Supplement: Supplementary file 1 [file plants-12-00407-s001.zip › plants-2086844-supplementary.pdf]

Table S1A. Allelic variants matrix showing the homozygous (2), heterozygous (1) and the absence (0) of the allele for the examined *loci*.

Accession	P1_155	P1_156	P1_164	P2_153	P2_157	P2_162	P2_165	P2_168	P3_180	P3_184	P3_186	P3_190	P3_192	P3_194	P4_282	P4_288	P4_291	P5_294	P5_304	P5_308
CVF1.1	1	1	0	0	0	2	0	0	0	0	0	0	2	0	0	0	0	0	1	1
CV1	1	1	0	0	0	0	2	0	0	0	0	2	0	0	0	2	0	0	0	2
CV4	1	1	0	0	0	0	1	0	0	0	0	0	2	1	0	2	0	0	1	1
CI1	1	1	0	0	0	0	1	0	0	0	0	2	0	0	0	2	0	0	0	2
CVF1.2	1	1	0	0	0	2	0	0	0	0	0	0	1	1	0	2	0	0	1	0
CV5	1	1	0	0	0	0	2	0	0	0	0	0	0	1	0	2	0	0	1	1
CV6	1	1	0	0	0	0	2	0	0	0	0	0	2	0	1	0	0	0	0	2
CV7	1	1	0	0	0	1	1	0	0	0	0	1	1	1	0	2	0	0	0	2
CVF1.3	1	1	0	0	0	0	1	0	0	0	0	0	1	0	0	2	0	0	0	2
CV8	1	1	0	0	0	1	1	0	0	0	0	2	2	1	0	2	0	0	0	2
CVF1.4	1	1	0	0	0	2	0	0	0	0	0	0	1	1	0	2	0	0	1	1
CVF1.5	2	0	0	0	0	2	0	0	0	0	0	0	0	2	0	2	0	0	0	2
CV9	2	0	0	0	0	0	1	0	0	0	0	0	2	0	0	2	0	0	1	1
CV10	1	0	0	0	0	0	0	0	0	0	0	0	2	0	0	1	1	0	0	2
CVF1.6	1	1	0	0	0	0	1	0	0	0	0	2	0	0	0	2	0	0	0	2
BRF1.1	1	1	0	0	0	2	0	0	0	0	0	0	1	0	0	1	0	0	0	2
BR	1	1	0	0	0	2	0	0	0	0	0	0	1	1	1	1	0	0	0	2
CVF1.7	0	2	0	0	0	1	1	0	0	0	2	0	2	0	0	2	0	0	1	2
CV11	1	1	0	0	0	0	1	0	0	0	0	0	2	0	0	2	0	0	0	2
BR1	1	1	0	0	0	2	0	0	0	0	0	0	2	0	0	2	0	0	1	1
BR2	1	1	0	0	0	2	0	0	0	0	2	0	0	0	0	1	1	0	2	0
CV2	1	0	0	0	0	0	2	0	0	0	0	2	0	0	0	2	0	0	0	2
BR3	0	2	0	0	0	0	0	0	0	0	0	0	2	0	1	0	1	0	0	2
BR4	1	1	0	0	0	0	1	0	0	0	0	0	2	0	0	1	1	0	0	2
BRF1.2	1	1	0	0	0	2	0	0	0	0	0	0	0	1	0	2	0	0	0	2
BR5	1	1	0	0	0	2	0	0	0	0	0	1	1	0	0	2	0	0	0	2
CV3	1	1	0	0	0	2	0	0	0	0	0	0	2	0	0	2	0	0	0	2
BR6	0	1	0	0	0	1	1	0	1	0	0	0	1	0	0	2	0	0	0	2
BR7	0	2	0	0	0	2	0	0	0	0	1	0	0	0	0	1	1	0	0	1
BR8	0	2	0	0	0	2	0	0	0	0	0	2	0	0	0	1	1	0	0	2
BR9	0	1	0	1	0	1	0	0	0	0	0	0	0	0	0	0	2	0	1	1
BU1	0	0	0	0	2	0	0	0	0	2	0	0	0	0	1	0	1	2	0	0
BU2	0	0	0	0	1	0	0	0	0	0	0	0	0	0	0	0	0	0	0	0
BY1	1	1	0	0	0	2	0	0	0	2	0	0	0	0	0	0	2	1	0	0
BM	0	1	0	0	0	0	0	1	0	0	1	0	0	0	0	0	1	0	0	0
BU3	0	0	0	0	2	0	0	0	0	0	0	0	0	0	2	0	0	2	0	0
BU4	1	1	0	1	0	1	1	0	1	2	2	0	0	0	1	1	1	0	0	2
BY2	0	2	0	0	0	2	0	0	0	2	0	0	0	0	0	2	0	0	0	0
BV	0	1	0	0	0	1	0	0	0	0	0	0	0	0	1	0	0	0	0	0
